# A modernização do ensino na Escola Paulista de Medicina e a Fundação Rockefeller, 1956-1962

**DOI:** 10.1590/S0104-59702024000100074

**Published:** 2025-01-10

**Authors:** Ricardo dos Santos Batista

**Affiliations:** i Professor, Programa de Pós-graduação em História/ Universidade do Estado da Bahia Alagoinhas – BA – Brasil kadobatista@hotmail.com

**Keywords:** Escola Paulista de Medicina, Circulação do conhecimento, Fundação Rockefeller

## Abstract

Analisa-se a circulação do conhecimento científico entre Brasil e EUA, a partir dos financiamentos da Fundação Rockefeller à Escola Paulista de Medicina para modernizar o ensino médico. O marco cronológico inicial escolhido é 1956, quando a fundação inaugurou o financiamento à instituição brasileira, e o final é 1962, ano-limite para gastar os valores disponibilizados. As fontes utilizadas são dossiês coletados no Rockefeller Archive Center e analisados a partir do paradigma indiciário. Conclui-se que, quando a agência filantrópica decidiu financiar a escola, esta já integrava uma rede de circulação internacional de conhecimentos, e que a modernização do ensino médico na instituição foi marcada por investimentos nas ciências básicas, em clínica médica, e com a defesa de departamentos em tempo integral.

Em *Relocating modern science: circulation and the construction of knowledge in South Asia and Europe, 1650-1900*, o pesquisador indiano Kapil Raj (2007) afirma que, nas últimas décadas, a alegada unidade das práticas modernas de conhecimento em todo o espaço europeu tem sido convincentemente demolida. No lugar de uma “ciência moderna” única, agora se aceita o fato de que existem muitas tradições e dinâmicas de conhecimento nacionais e locais espalhadas pela maior parte do norte e do oeste da Europa, com agendas e influências intelectuais modernas diversas. Em sua interpretação, questiona amplamente conceitos como “centro”, que produziria o conhecimento a ser transplantado, e “periferia” vista como lócus de recepção passiva desse saber (p.6). O autor se contrapõe às ideias unidirecionais de disseminação e ao binarismo presente em interpretações dicotômicas e propõe a utilização do conceito de circulação não como mera transmissão ou comunicação de ideias, mas como processos de encontro, poder, resistência, negociação e reconfiguração que ocorrem na interação entre culturas, por meio de indivíduos “mediadores” ou agentes locais, que atuam nas “zonas de contato” ou nas interações com outras culturas. Pautado nesses pressupostos, este texto tem como objetivo analisar a circulação do conhecimento científico entre Brasil e EUA, entre 1956 e 1962, por intermédio do financiamento da Fundação Rockefeller (FR)^1^ à Escola Paulista de Medicina (EPM).

Antes de a Organização Mundial da Saúde ser fundada, em 1948, a International Health Divison (IHD) da Fundação Rockefeller foi a agência filantrópica mais importante do mundo no trabalho em saúde pública. Desde seu surgimento, lhe atribuíram diferentes nomes: International Health Comission, entre 1913 e 1916; International Health Board (IHB), de 1916 a 1927; e, de 1927 até seu fechamento, em 1951, funcionou como IHD (Farley, 2004, p.2). Os membros do IHB seguiram os princípios filantrópicos de John D. Rockefeller, milionário norte-americano que investiu na agência internacional com o dinheiro oriundo de exploração, refino e comércio de petróleo, e que defendia a ideia de que a filantropia não poderia ser confundida com a caridade. Ela deveria ser encarada como um investimento oferecido a agências governamentais, e não a indivíduos, com duração limitada, para não se tornar dependência, destinada a organizações comprometidas com a continuidade do trabalho quando o auxílio terminasse (Farley, 2004, p.3-5). Especialmente após o fechamento da IHD, a FR diminuiu os recursos ofertados para projetos de saúde pública e passou a investir mais amplamente em ciências básicas ou de laboratório.

Segundo Gabriela Marinho (2004), o apoio da FR a pesquisadores da EPM é um processo ainda insuficientemente analisado e bem menos conhecido que a presença da agência internacional na Universidade de São Paulo (USP), por exemplo. Ela também afirma que, embora a instituição tenha sido decisiva na constituição de um padrão de pesquisa, referências escassas e pontuais não conduziram, ainda, à formulação de análises mais consistentes sobre os impactos na sua produção científica e na construção de um modelo de excelência relacionado a agendas específicas. Dessa forma, esta análise contribui para a ampliação do conhecimento sobre a relação entre a agência filantrópica internacional e a EPM, além de observar o conhecimento produzido na instituição brasileira como parte de uma rede de conhecimentos internacionais. O texto se aproxima de interpretações realizadas em livro organizado por Schneider (2002), para as quais os interesses filantrópicos da FR tentaram se reformar em meio ao complexo jogo de influências locais e mais amplas.

Os documentos utilizados para a escrita do artigo foram coletados *in loco* no Rockefeller Archive Center, em maio de 2019, e contribuem para a compreensão da modernização do ensino médico no Brasil como um processo complexo e não linear. Entre as instituições financiadas pela FR em São Paulo com essa finalidade, e cuja documentação foi consultada e digitalizada, destaca-se a Faculdade de Medicina de Ribeirão Preto. Esse talvez seja o caso mais emblemático no Brasil na década de 1950, visto que, desde sua criação, o esperado era que se tornasse “a Johns Hopkins da América Latina, o modelo de escola de medicina moderna” (Barron, 17 dez. 1952a, p.1),^2^ em menção explícita à primeira universidade norte-americana apoiada pela FR nos inícios do século XX (Marinho et al., 2024).

Destaca-se que também foram encontradas fontes sobre a EPM. Para as duas instituições, há dossiês relativos aos financiamentos oferecidos (*grants*), compostos por cartas e correspondências; dossiês específicos sobre os materiais comprados para departamentos, laboratórios, bibliotecas etc., e sobre os profissionais que receberam bolsas para estudo no exterior, com o objetivo de ajudar a modernizar o ensino. Ao longo da sua história, no século XX, a FR acreditou que poderia formar lideranças ou “elementos-chave” que propagariam o conhecimento adquirido quando retornassem ao país de origem, e por isso implementou um duradouro programa de bolsas, conforme demonstram os estudos de trajetórias de bolsistas realizados por Batista (2019a, 2019b, 2020a, 2020b, 2020c, 2023); Batista e Silva (2020); Batista e Ferreira (2021, 2023); Batista, Porto e Ferreira (2023); e Batista e Mota (2021). Em outra perspectiva de análise, prosopográfica, é possível encontrar trabalhos como os de Korndörfer (2016, 2019) e Korndörfer e Ramacciotti (2021).

As fontes utilizadas neste texto são compostas por dossiês sobre a EPM que agregam, além dos documentos citados, cartilhas de divulgação do trabalho desenvolvido pela instituição antes do apoio da agência internacional. O método de análise recorrido é o onomástico, que prioriza o “nome” como elemento-chave na pesquisa de arquivo, por meio do paradigma indiciário, que evidencia os pequenos detalhes das fontes como sinais importantes para a análise (Ginzburg, 1989a, 1989b).

## Modernização do ensino médico e investimento em ciências básicas

A modernização do ensino médico foi uma questão importante para a FR ao longo de todo o século XX. Segundo Elizabeth Fee (2016), no final do século XIX, a saúde pública foi institucionalizada nas secretarias municipais e estaduais de saúde dos EUA, mas poucos sanitaristas tinham formação especializada na área. Alguns eram médicos, outros engenheiros, advogados, químicos ou biólogos. Geralmente eles recebiam cargos de meio período, pagos por patrocínio político, e poderiam ser promovidos ou removidos de suas funções com base em alianças políticas e amizades pessoais. Contudo, um novo modelo de treinamento médico emergente nos EUA consolidou recursos em escolas de elite – a partir do relatório produzido por Abraham Flexner, em 1910 –, que mudaram a forma de treinamento médico no país. Entre as transformações estabelecidas estavam a recomendação para experiência clínica em hospitais de ensino controlados por um corpo docente em tempo integral e a familiaridade dos estudantes com o laboratório (Cueto, Palmer, 2016).

Em sintonia com o Relatório Flexner, em 1914, um pequeno grupo de líderes de saúde pública reunido a convite do General Education Board da FR identificou a necessidade de criar instituições formais para treinar profissionais de saúde (Fee, 2016). Progressivamente, o investimento da agência internacional em escolas de saúde pública e medicina se ampliou para diferentes lugares do globo. Como mencionado, a primeira delas foi a Johns Hopkins School of Hygiene and Public Health (Fee, 2016), seguida pela Faculdade de Medicina e Cirurgia de São Paulo (FMCSP) (Batista, Mota, 2021). Entre muitos outros exemplos que poderiam ser citados estiveram os investimentos em Edimburgo, onde a tradição e o individualismo eram altamente valorizados, mas considerados pela FR um obstáculo à sua visão de reforma médica (Lawrence, 2005); e no leste da Ásia, onde houve financiamento para o Peking Union Medical College, por meio do China Medical Board da FR. Durante três décadas, a instituição passou a ser considerada “o berço da medicina moderna na China” (Ryan, Bullock, 2017, p.22).

Em relação à América Latina, entre 1913 e 1940, a FR estendeu seu interesse em financiar ciências no campo da fisiologia no Peru e na Argentina. Entre os argentinos, Bernardo Houssay, premiado com o Nobel em 1947, dirigiu o Instituto de Fisiologia da Universidade de Buenos Aires. De acordo com Cueto (1994), a atuação da FR na América Latina teve três dimensões. Primeiro, ao conceder bolsas de estudo para estudantes de medicina nos EUA, desencorajou a preferência dos médicos latino-americanos por pós-graduação em países da Europa, especialmente em Paris. Em segundo lugar, porque a FR tinha profundas dúvidas sobre as escolas médicas tradicionais, promoveu o estabelecimento de novos centros educacionais e de pesquisa independentes dessas instituições. E, em terceiro lugar, a agência deu dinheiro a pesquisadores destacados nas ciências básicas. A doação de equipamentos de primeira linha para instituições dispostas a garantir vagas para fisiologistas em tempo integral tornou-se um dos principais objetivos da FR (Cueto, 1994).

O investimento da Rockefeller nas denominadas ciências básicas no Brasil foi analisado por Paloma Porto (2021), que estudou as dinâmicas nas políticas de financiamento científico no Instituto de Ciências Biológicas (ICB) da Universidade Federal de Minas Gerais (UFMG). A autora discute o programa fomentado pela agência filantrópica internacional com o objetivo de treinar professores em ciências básicas, comandado na América Latina por Robert Briggs Watson. A FR estimulou tendências cognitivas de escala micro nas pesquisas científicas do ICB, com ênfase no manejo de equipamentos modernos e espaços laboratoriais, redirecionando, cada vez mais, seus investimentos para uma “molecularização” das ciências (Porto, 2021).

No início do século XX, funcionários da FR haviam visitado várias faculdades de medicina, e se assustaram, porque a maior parte dos livros de suas bibliotecas era em francês. Por volta de 1950, eles voltaram a visitar as faculdades e perceberam que a maioria dos livros era de língua inglesa (Cueto, 2021). Outras mudanças importantes, operadas ao longo da primeira metade do século XX, foram a integração do laboratório com a clínica e a ascensão da genética, da biologia molecular e da biofísica na América Latina. Além disso, percebeu-se a existência de uma retórica ideológica de legitimação científica em um período marcado pelos esforços dos governos norte-americanos em conter a proliferação do comunismo (Cueto, 2021).

A modernização da educação médica proposta pela FR, na segunda metade do século XX, ocorreu em meio ao conflito da Guerra Fria, no qual a cooperação técnica internacional foi um elemento de destaque. De acordo com Onofre Santos Filho (2005), em meio à bipolaridade EUA *versus* URSS, as ideias de desenvolvimento e de modernização encontraram campo profícuo para sua propagação. No Ponto IV do discurso de posse do presidente norte-americano Harry Truman (1945-1953), por exemplo, ele mencionou um novo padrão para a relação com os Estados denominados “subdesenvolvidos” e “desenvolvidos”. A partir desse modelo, as bases da relação entre Norte e Sul deveriam ocorrer a partir da oferta de auxílio, por parte dos países desenvolvidos, para que os países subdesenvolvidos deixassem a condição de miséria e de pobreza em que viviam. Os países ricos, responsáveis pela condução dessa alteração, não teriam recursos materiais abundantes passíveis de ser transferidos aos Estados pobres, mas disporiam de conhecimentos tecnológicos que poderiam ser repassados. Nessa lógica, bastaria aos últimos seguir os passos dos primeiros, que conseguiriam o conhecimento científico e tecnológico para se transformar e se livrar da pobreza e da ignorância, únicas causas do atraso em que se encontravam. Ainda de acordo com Santos Filho (2005), além de atender aos parâmetros do novo ordenamento do pós-guerra e do conflito bipolar, essa concepção de desenvolvimento permite identificar os agentes de transformação: as organizações internacionais recém-criadas, os Estados nacionais e as elites modernizantes.

Em “Educational reform, modernization and development: a Cold War transnational process”, Óscar J. Martín García e Lorenzo Delgado Gómez-Escalonilla (2020) afirmam que os EUA foram uma força líder por trás dos processos de reforma educacional na Guerra Fria. Na década de 1960, o governo norte-americano começou a demonstrar mais interesse pela educação nas suas relações com os países denominados “periferia” e “semiperiferia” global. Em setembro de 1961, o relatório “Políticas e programas educacionais e culturais para a década de 1960” reuniu propostas de vários grupos de trabalho reunidos pela administração de John Kennedy (1961-1963), com o intuito de elaborar “uma filosofia e objetivos para as atividades educacionais, culturais e científicas para a década de 1960, no que se referia a setores governamentais e privados. De acordo com o relatório, a educação foi um ingrediente básico das fases iniciais do desenvolvimento econômico” (García, Gómez-Escalonilla, 2020, p.14).

Os autores ainda destacam que o arranque rumo à modernização dos países considerados atrasados envolveria, a partir de sistemas educativos modernos, formação para criar capital humano com capacidades técnicas necessárias para resolver os problemas do subdesenvolvimento, especialmente em programas internacionais de educação, cultura e ciência. No entanto, os programas educacionais não foram realizados apenas por atores governamentais. A Organização das Nações Unidas para a Educação, Ciência e Cultura, a Organização para a Cooperação e Desenvolvimento Econômico e o Banco Mundial, por exemplo, também foram interlocutores de reforma educacional. E, nesse contexto, a FR intensificou suas ações de financiamento da modernização do ensino médico, caso observado na EPM.

## A EPM e os departamentos em tempo integral

A criação da EPM ocorreu em um contexto propício à expansão do ensino médico em São Paulo. Desde 1891, a legislação paulista já havia previsto a criação de uma escola de Medicina, mas a FMCSP só foi fundada em 1913 (Mota, 2005). A FMCSP passou por transformações na reforma Pedro Dias, em 1924, e na reorganização promovida pelo decreto 5.351 de 16 de janeiro de 1932, com o objetivo de se adaptar às mudanças impostas pela reforma educacional Francisco Campos. Em 1933, o número de inscritos para o exame vestibular da FMCSP foi elevado, e, mesmo que 119 inscritos tivessem obtido a média para ingressar no curso pré-médico, só havia setenta vagas. De acordo com Márcia Regina Barros da Silva (2003), houve insatisfação por parte dos excedentes desse exame, que passaram a solicitar a ampliação das vagas. Contudo, os aspirantes a médico não imaginavam a intenção de outro grupo, formado por professores que desejavam um novo espaço para o ensino superior de medicina em São Paulo. A convergência dos interesses entre os dois grupos teria sido fundamental para a criação da EPM.

**Figura 1:**
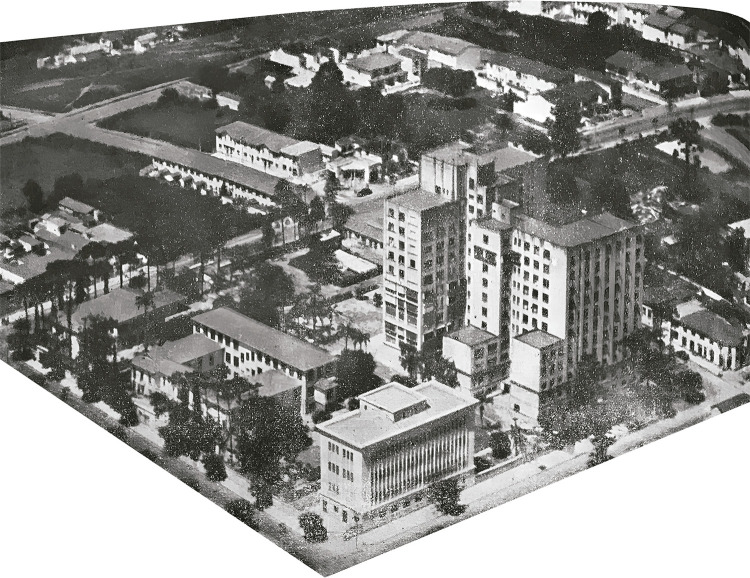
: Escola Paulista de MedicinaMedicina (Fonte: EPM, 1951, p.12)

Assim, em 26 de junho de 1933, estava criada a nova instituição médica paulista (Figuras 1 e 2). Seus fundadores eram vinculados a três grandes áreas de atuação: à prática docente, na Santa Casa de Misericórdia; à prática clínica nos serviços sanitários do estado de São Paulo; e às atividades realizadas em institutos de pesquisa (Silva, 2003). A produção historiográfica atual afirma que a história da EPM pode ser dividida em duas fases. A primeira compreende a instalação do curso médico, em 1933, até o início da construção do Hospital das Clínicas. A segunda se inicia com a formatura da primeira turma, em 1938, e vai até a federalização da Escola, em 1956 (Silva, 2003). Este artigo tem como objetivo auxiliar na compreensão de aspectos relativos ao que pode ser considerado uma terceira fase, caracterizada pelo amplo financiamento oferecido à EPM pela FR, entre 1956 e 1962.

**Figura 2:**
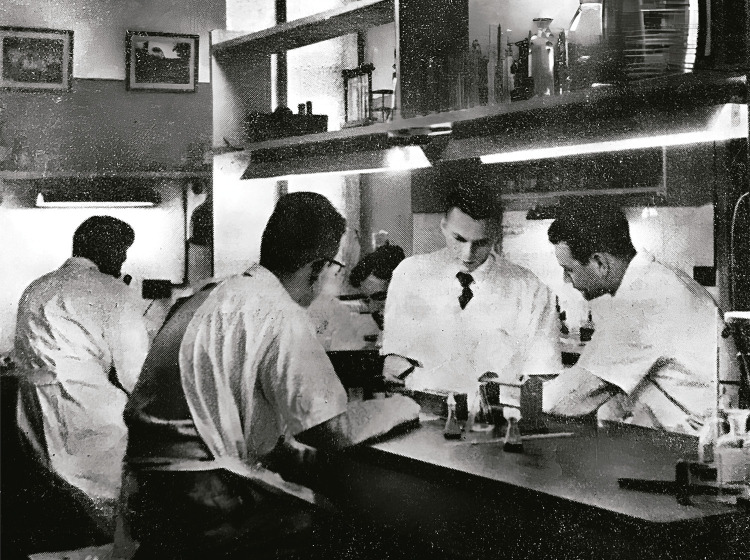
: Estudantes na Escola Paulista de Medicina (Fonte: EPM, 1951, p.17)

Nos anos que antecederam o financiamento da FR à instituição brasileira, a União Cultural Brasil-Estados Unidos, afirmando cumprir com seu papel de promover conhecimento e entendimento entre os dois países, escreveu ao *staff* da agência filantrópica com o objetivo de chamar a atenção para uma organização médica paulista, que por sua eficiência e honestidade científica merecia ser mais conhecida: a EPM (Amorim, 5 jul. 1951). A correspondência redigida pelo diretor-presidente, Rone Amorim (5 jul. 1951, p.1), afirmava que a escola já havia alcançado reputação invejável, por manter altos padrões de ensino, e, por isso, sugeria a visita de um membro da Rockefeller, quando estivesse em São Paulo, o que garantiria um contrato “mutuamente vantajoso”. Em resposta, Robert Morison, diretor assistente da Divisão de Ciências Naturais da FR, agradeceu pela correspondência, informou que alguns membros da agência já conheciam a escola e que, em breve, esperava que pudessem fazer uma nova visita a São Paulo.

Um dos membros designados para essa observação foi Oliver Jones (27 out. 1952), que emitiu opinião contrária ao financiamento da EPM. Ele escreveu para Harry Miller Jr. e demonstrou ter ficado bastante impressionado com as atividades de pesquisa em São Paulo, mas não se impressionou com a forma pela qual as escolas de medicina eram administradas. Ele duvidava que seria possível elevar o padrão de medicina ou a qualidade da clínica geral, a menos que algo drástico fosse feito sobre a “política de admissão na escola e a eliminação de alunos fracos após o segundo ou mesmo terceiro ano da faculdade de medicina” (Jones, 27 out. 1952, p.1). A principal crítica à Paulista de Medicina era que ela dependia muito das mensalidades dos estudantes para funcionar, e, como resultado, admitia turmas maiores do que poderia acomodar em suas estruturas física e de pessoal. Ao mesmo tempo, o fato de ser privada permitia uma atitude mais progressista em relação às nomeações e promoções do corpo docente (Jones, 11 nov. 1952).

Depois de pouco mais de um mês, outro membro do *staff* da FR, E.S. Guzman Barron, que também visitou a instituição, demonstrou entusiasmo com o diretor da EPM, Jairo Ramos, e com o professor de bioquímica Leal Prado. De acordo com Barron (17 dez. 1952b, p.1), este último, definido como um ex-fisiologista ansioso para se dedicar inteiramente à bioquímica, teria sido um dos principais fatores para a conversão do projeto em Ribeirão Preto de “uma espécie de creche paulista em uma moderna escola médica”. E, após ponderar as avaliações, a FR decidiu investir na EPM.

O primeiro financiamento (Grant RF 50652) foi concedido para o desenvolvimento da instituição paulista, em abril de 1956, com a soma de 105 mil dólares a ser utilizada até 30 de junho de 1958, benefício posteriormente estendido até o mês de novembro. A concessão ocorreu depois que o doutor Bugher, do *staff* da FR, expôs um projeto que definia o auxílio para a formação profissional em educação médica e saúde pública, e também para pesquisa biológica e médica (Rockefeller Foundation, 4 abr. 1956). O objetivo do apoio da FR era integrar a EPM a seu programa básico para a melhoria do ensino médico e da pesquisa no Brasil, por meio do fornecimento de instalações de treinamento para a implementação de bolsas de estudos e de material de pesquisa:

Apesar dos recursos financeiros limitados e das instalações clínicas inferiores às da Faculdade de Medicina de São Paulo, a Escola Paulista tem, desde o início, a reputação de estar entre as melhores escolas médicas do Brasil. Embora careça em profundidade de corpo docente e pessoal técnico subordinado, particularmente, nos departamentos pré-clínicos, seus chefes de departamento e seus principais assistentes compõem um grupo de professores e pesquisadores igual ao de qualquer outra escola brasileira. Alguns desses professores são os representantes de ponta de suas disciplinas profissionais que o Brasil pode apresentar, e esta dotação proposta destina-se especialmente a apoiar esses departamentos (Rockefeller Foundation, 4 abr. 1956, p.2-3).

Apesar da denúncia relativa à carência no corpo profissional, antes mesmo do financiamento da FR, o corpo docente da EPM contava com indivíduos que se tornariam referências na produção científica nacional. Em 1951, trabalhavam na instituição André Dreyfus, na cátedra de Histologia e Embriologia Geral; Antônio Carlos Pacheco e Silva, na Clínica Psiquiátrica; e Walter Pereira Leser, na cátedra de Higiene. O desenvolvimento institucional da EPM havia sido impulsionado com o apoio recebido do Departamento de Saúde do Estado, da Diretoria da própria EPM e, a partir de 1952, do Conselho Nacional de Pesquisas, o que contribuiu para que os laboratórios de Farmacologia e Bioquímica lograssem êxito em suas atividades. Em relação aos estudantes, no final do ano letivo de 1954, a instituição já havia formado 1.366 médicos, sendo 402 da cidade de São Paulo, 494 do estado de São Paulo, 82 dos estados de Paraná, Rio Grande do Sul, Minas Gerais e Distrito Federal, 34 de outros estados diversos e 17 de países estrangeiros como Canadá e EUA (Rockefeller Foundation, 4 abr. 1956).

A produção de um material comemorativo do funcionamento entre 1948 e 1953 apresenta dados que auxiliam a identificar alguns dos profissionais que compunham a rede de produção e circulação do conhecimento científico na EPM (Quadro 1).

**Quadro 1:** : Quadro de pessoal dos laboratórios de Bioquímica e Farmacologia, 1948-1953

Nome	Formação
José Ribeiro do Valle	Professor catedrático de farmacologia, antigo assistente do Instituto Butantan, médico do Departamento de Saúde do Estado de São Paulo
José Leal Prado	Professor catedrático de química fisiológica, antigo assistente do Instituto Butantan, médico do Departamento de Saúde do Estado de São Paulo
Zuleika Pentone Picarelli	Química pela Faculdade de Filosofia, Ciências e Letras da Universidade de São Paulo, primeira assistente da cadeira de farmacologia com dedicação exclusiva graças a auxílio do Conselho Nacional de Pesquisa
Eline S. Prado	Química pela Faculdade de Filosofia, Ciências e Letras da Universidade de São Paulo, mestre em ciências pela Universidade de McGill, no Canadá, primeira assistente de química fisiológica, com dedicação exclusiva graças a auxílio do Conselho Nacional de Pesquisas
Antônio Cechelli de Mattos Paiva	Médico pela Escola Paulista de Medicina, segundo assistente da cadeira de química fisiológica, com dedicação exclusiva, recipiendário, em 1953, da Bolsa de Estudos Doutor Joaquim Libânio Leite Ribeiro
Catharina M. W. Brandi	Química pela Faculdade de Filosofia, Ciências e Letras da Universidade de São Paulo, terceira assistente da cadeira de química fisiológica, bolsista dos Laboratórios Lorderle
Bella Regina Kupper	Farmacêutica, assistente extranumerária, com dedicação exclusiva

Fonte: Rockefeller Foundation (1948-1953).

Em 1954, o currículo da EPM também havia sido drasticamente reorganizado. As mudanças foram aprovadas pelo Conselho Nacional de Educação e entraram em vigor em 1955. Os membros da FR opinavam que a organização da Escola se comparava positivamente à das escolas dos EUA, do ponto de vista do ensino, e as principais discrepâncias eram impostas pela legislação brasileira, a exemplo da necessidade de educação pré-médica e certos requisitos legais (Rockefeller Foundation, 4 abr. 1956). Três cadeiras de ciências básicas eram ocupadas por professores e pesquisadores de destaque em tempo integral: a de bioquímica, por José Leal Prado, a de farmacologia, por José Ribeiro do Valle, e a de microbiologia, por Otto Bier. Cada uma dessas cátedras deveria receber do Conselho Nacional de Pesquisas, em 1956 e posteriormente, verbas para a complementação dos salários de auxiliares e técnicos profissionais, de modo que os quadros desses três departamentos funcionassem em regime de tempo integral.

A partir de 1 de março de 1956, duas cátedras (bioquímica e farmacologia) passaram para um novo prédio destinado ao ensino, à pesquisa e ao uso conjunto de equipamentos. A cátedra de patologia, também ocupada por um professor titular, Moacyr de Freitas Amorim, solicitou apoio do Conselho Nacional de Pesquisas, em 1956, e construiu mais um edifício para abrigar as disciplinas relacionadas àquela área (Rockefeller Foundation, 4 abr. 1956). Entre 1957 e 1958 esse departamento foi expandido e ocupava salas completamente recondicionadas e reequipadas (Rockefeller Foundation, 29 set. 1959).

Os investigadores dos laboratórios da EPM mantinham intenso contato com pesquisadores nacionais e estrangeiros, por meio de troca de correspondências, permuta de bibliografia e visitas recíprocas. Entre as instituições com as quais eles se comunicavam estavam escolas e faculdades da Universidade de São Paulo, a Faculdade de Medicina de Belo Horizonte (Laboratórios de Anatomia, Patologia, Farmacologia e Química Fisiológica), a Faculdade de Medicina de Recife (Instituto de Fisiologia Álvaro Ozório de Almeida), a Faculdade de Medicina do Paraná (Laboratórios de Fisiologia e Farmacologia), a Faculdade Nacional de Medicina, o Instituto Oswaldo Cruz, o Instituto Butantan, entre outros (Rockefeller Foundation, 1948-1953).

Os laboratórios da EPM já haviam sido visitados por R.A. Lambert, Alan Gregg, W.W. Oliver e Harry Miller Jr., todos membros do *staff* da FR; Luiz F. Leloir e R. Caputo, de Buenos Aires; E. Rothlin, da Basilea; A.L. Tatum, de Madison, e A. de Barbieri, de Milão. Por fim, entre os 17 pesquisadores que fizeram conferências na EPM estiveram nomes como Bernardo A. Houssay, diretor do Instituto de Biologia e Medicina Experimental de Buenos Aires; Frank G. Youn, professor de bioquímica na Universidade de Cambridge; e J.M. Carlisle, diretor da Divisão Médica da Merck, EUA, além de Earl Walker, professor de neurocirurgia da Universidade de Johns Hopkins (Rockefeller Foundation, 1948-1953).

É possível afirmar, portanto, que pesquisadores da EPM se relacionavam com integrantes de grandes centros de conhecimento médico no mundo e já despontavam em uma complexa rede de trocas de conhecimento, desde o início dos anos 1950. O financiamento proposto pela FR, em 1956, seguia o diagnóstico realizado por Steven Palmer (2015) sobre o modo de ação da agência internacional, para a qual o interesse estava em instituições e países que já desenvolviam algum tipo de ação na área em que se pretendia investir. Desde o seu surgimento, a FR apoiava instituições governamentais que pudessem, após algum tempo de financiamento, assumir a responsabilidade pela ação desenvolvida e servir como exemplo para a replicação do seu modelo (Marinho, 2013).

**Figura 3:**
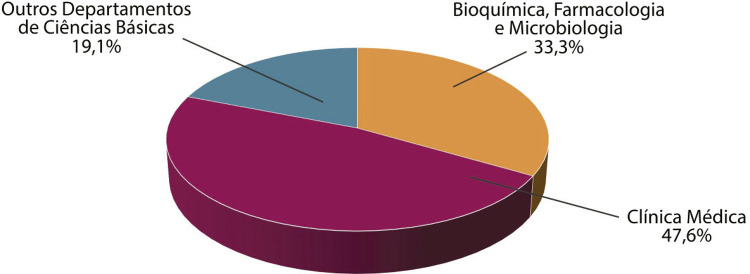
: Distribuição dos recursos, Grant RF 56052 (Fonte: Rockefeller Foundation, 4 abr. 1956, p.5)

O apoio da Rockfeller à EPM era destinado à compra de equipamentos de ensino e de pesquisa de graduação nos departamentos de Bioquímica, Farmacologia e Microbiologia, e a viabilizar o acolhimento de estudantes nacionais e estrangeiros na pós-graduação. Esperava-se que os três departamentos se tornassem importantes centros de formação de professores das escolas médicas brasileiras, no âmbito de um projeto que, naquele momento, era financiado pela Coordenação de Aperfeiçoamento de Nível Superior (Capes) (Rockefeller Foundation, 4 abr. 1956). A recomendação era no sentido de que o recurso cedido pela FR fosse dividido da seguinte maneira: 52,4% para ciências básicas (sendo 33% exclusivamente para bioquímica, farmacologia e microbiologia) e 47,6% para clínica médica (Figura 3).

A distribuição do recurso mantinha certo equilíbrio entre as áreas financiadas, demonstrando as prioridades da FR e da EPM no processo de modernização do ensino. Como será possível observar, a clínica médica foi impulsionada, especialmente, pelo desempenho obtido pelo professor Jairo Ramos.

Em 3 de dezembro de 1958, novo auxílio, de seiscentos mil dólares (Grant RF 58217), foi aprovado com o objetivo de garantir a consolidação e continuação de desenvolvimento da EPM, com a mudança de todos os departamentos para um regime de ensino e pesquisa em tempo integral. O valor deveria ser gasto em período mínimo de três anos e máximo de cinco anos, a partir de 1 de janeiro de 1959 (Rockefeller Foundation, 3 dez. 1958). Os bons resultados do investimento na EPM fizeram com que nova dotação, de U$ 633.500,00 (Grant RF 58...), fosse autorizada, em 1959, com o mesmo objetivo de consolidar o desenvolvimento da escola, até 31 de dezembro de 1961:

O notável progresso da escola durante os dois últimos anos não foi previsto. Este progresso sugere que, com mais, e mais amplo apoio a escola pode desenvolver uma posição de liderança preeminente da educação médica brasileira. Essa perspectiva parece por si só, e em relação ao desenvolvimento do programa no Brasil, justificar a consideração favorável do pedido da administração da escola, endossado ao Ministério da Educação Federal por tal apoio (Rockefeller Foundation, 29 set. 1959, p.2).

É interessante observar que o primeiro orçamento oferecido à EPM foi uma aposta no seu potencial de desenvolvimento, mas não havia dimensão exata das implicações futuras da ação. A instituição havia sido incorporada ao sistema educacional federal fazia apenas dez semanas, e o restante de 1956 foi utilizado para transferir seu patrimônio à União, exceto o Hospital São Paulo (seu hospital das clínicas), e para cumprir outras formalidades legais.

Para a FR, a qualidade dos docentes da EPM foi melhorada por bolsas de estudo e bolsas de viagem. O professor de bioquímica, por exemplo, passou um ano na França e nos EUA, e seu principal assistente passou dois anos nos EUA como bolsista. Suas esposas também eram formadas em bioquímica, atuavam como auxiliares no departamento, os acompanharam ao exterior e, de certa forma, acreditava-se que tivessem sido beneficiadas profissionalmente com a viagem (Rockefeller Foundation, 29 set. 1959). Não raro, as assimetrias de gênero, construídas social e culturalmente, impediam que mulheres esposas de cientistas fossem beneficiadas com uma bolsa própria. Restava a elas aproveitar a viagem como acompanhantes de seus maridos, tentar produzir conhecimentos e implementar, quando possível, agendas de pesquisas em instituições internacionais.

Ainda em relação a bolsistas da EPM, duas bolsas do programa de Educação Médica e Saúde Pública (MEPH) para assistentes do Departamento de Medicina estavam em vigência: em 1959, um bolsista retornaria após dois anos de treinamento, e o outro, após um. Por fim, a presença de oito bolsistas na escola com financiamento da Capes e da FR, grande parte nos departamentos de ciências básicas, os fortaleceu pedagogicamente, preparando-os para seus cargos no Brasil e no Chile (Rockefeller Foundation, 29 set. 1959).

O governo brasileiro também contribuiu com aproximadamente trezentos mil dólares, em 1957, e 303 mil dólares, em 1958. Ofereceu, ainda, aproximadamente cinco milhões de cruzeiros para completar o novo prédio do Departamento de Bioquímica e Farmacologia, reconstruir o de Microbiologia e iniciar o novo prédio da biblioteca (Rockefeller Foundation, 29 set. 1959). Contudo, os fundos do orçamento operacional forneciam apenas empregos de meio período para o corpo docente. Para viabilizar o emprego em tempo integral dos “elementos-chave” de pesquisa, o Conselho Nacional de Pesquisas destinou 384 mil cruzeiros, em 1957, e 923.500 cruzeiros, em 1958.

Um departamento que se manteve sob a expectativa da FR durante todo o financiamento foi o de Clínica Médica. Esperava-se não só melhorar o ensino de graduação e pós-graduação, mas também fornecer equipamentos de pesquisa médica, como o de diagnóstico laboratorial para o uso dos estudantes nos casos médicos que lhes fossem atribuídos. Jairo Ramos, professor e chefe daquele departamento, era considerado pelo *staff* da agência destacado clínico e educador, também ex-diretor da escola. Acreditava-se que dois eventos ajudaram a cristalizar o papel desempenhado por ele na reforma da educação médica paulista: o Seminário de Educação Médica ocorrido em Ribeirão Preto, em 1956, e o contato com a Faculdade de Medicina de Cáli, em expedição da EPM que ele mesmo liderou.

Por fim, o contato frequente de Ramos com vários membros da FR, como Dana W. Atchley, Clifford Grullee, Robert Shank, Robert Glazer, George Reader e William B. Wendel, desde 1956, foi útil para formular planos para a EPM (Rockefeller Foundation, 29 set. 1959). Afirmava-se sobre Ramos que, com outros elementos progressistas da educação médica brasileira, talvez em maior medida do que qualquer outra pessoa, ele foi responsável por romper com a tradição no esforço de modernizar a educação médica no Brasil. Ramos e sua equipe básica de assistentes, oito dos quais foram treinados nos EUA, gastaram mais da metade do seu tempo ensinando (Rockefeller Foundation, 4 abr. 1956).

Os principais aspectos dos planos realizados por Jairo Ramos para a EPM, com o auxílio dos médicos da FR, eram: a criação de ciência básica completamente em tempo integral e o estabelecimento de tempo integral geográfico nos principais departamentos clínicos. Segundo Voltarelli (1997, p.152), nessa perspectiva, a maioria dos docentes receberia remuneração adicional por serviços assistenciais prestados ao hospital universitário e teria oportunidade de atender doentes privados ou conveniados e de dar plantões remunerados sem se afastar das suas instituições, o que configurava o regime de tempo integral geográfico.

Ainda sobre esse regime de trabalho, de acordo com Martins (2000), a Faculdade de Medicina de Ribeirão Preto da Universidade de São Paulo foi a primeira a ser criada no interior do país, com recursos totalmente públicos, e exigiu-se de todos os docentes a dedicação integral como forma de fomentar a pesquisa. Na época, já se reconhecia a pouca atração exercida pelos salários em áreas de aplicação, por isso se permitiu o exercício da clínica privada, de forma controlada e tempo semanal determinado, nas dependências da própria unidade (do total de 750 leitos do hospital associado, apenas 12 ou 1,6% se destinavam a essa finalidade). Seria então a chamada dedicação exclusiva geográfica ou tempo integral geográfico. Ofereceu-se ainda moradia gratuita, além da aposentadoria integral.

Em 1959, o Departamento de Clínica Médica da EPM contava com 18 médicos. Dez deles, incluindo Jairo Ramos e dois professores associados, eram remunerados em regime de tempo parcial, e os oito restantes eram professores voluntários, com nomeações pontuais no corpo docente. Considerava-se que cinco desses oito funcionários voluntários e não remunerados estavam “entre os melhores jovens da educação médica brasileira, quatro formados nos EUA, e um, o cardiologista, no Instituto de Cardiologia do México” (Rockefeller Foundation, 29 set. 1959, p.6). Ramos propunha assegurar nomeações permanentes para esses cinco homens.

Desejava-se, ainda, o desenvolvimento de um programa de treinamento para preparar o corpo técnico no Brasil e no exterior; completa reorganização da biblioteca médica e a construção de instalação especial para abrigá-la, em 1959-1960; a construção de um prédio para ciências fisiológicas, em 1960-1961, e a reforma dos espaços existentes para a expansão de departamentos associados à morfologia (Rockefeller Foundation, 29 set. 1959).

De todos esses aspectos, o que parecia ter maior destaque era a adoção do trabalho em tempo integral:

O tempo integral geográfico já foi colocado em prática na Escola Estadual de Medicina de Ribeirão Preto e em um departamento de Medicina Clínica da Faculdade de Medicina de Salvador (Bahia) em regime de tempo integral (dedicação exclusiva). Mas esta é a primeira vez no Brasil que uma Faculdade Federal de Medicina se propõe a estabelecer, o mais rápido possível, geografia em tempo integral em todos os seus departamentos clínicos. Somente por meio do estabelecimento desta disciplina administrativa pode a proposta do ensino nessa escola ser concretizada; essa reforma é essencial: é fundamental para qualquer melhoria real do ensino médico nessa ou em qualquer escola do Brasil (Rockefeller Foundation, 29 set. 1959, p.8).

A experiência desenvolvida na EPM empolgava o *staff* da FR, entre outros motivos, porque aquela era uma instituição federal, o que parecia oferecer bases mais seguras para a realização do projeto. A Faculdade de Medicina de Ribeirão Preto pertencia ao estado de São Paulo, e, na Faculdade da Bahia, o tempo integral geográfico foi implantado apenas em um departamento, o que não atendia satisfatoriamente aos anseios da modernização do ensino em uma instituição médica.

O conceito de reforma em clínica médica proposto pela EPM era considerado simples: os alunos receberiam formação abrangente na área, o mais cedo possível no currículo, e deveriam permanecer sob a supervisão de professores competentes durante todo o contato com os pacientes. Afirmava-se que a maioria dos estudantes de medicina brasileiros não tinha ocupação após o turno único da aula, ficava sem rumo depois do meio-dia, entregue a seus próprios planos, e que sua instrução, mesmo na melhor das circunstâncias, tendia a ser fragmentária, carente de coordenação, ineficiente e ineficaz (Rockefeller Foundation, 29 set. 1959).

A EPM se propunha a iniciar o treinamento clínico dos estudantes, no hospital, um ano antes no currículo em relação ao que ocorria em outras escolas. De acordo com o programa de ensino, no terceiro ano seriam ministradas 120 horas (em 15 semanas) de métodos de diagnóstico físico em medicina, cirurgia, neurologia e pediatria, com 100 horas (10 semanas) de desempenho e interpretação de testes clínicos de laboratório. O quarto ano traria o mínimo de 660 horas (em 15 semanas) de estágio clínico em medicina geral, incluindo neurologia e pediatria, seguido de 15 semanas de cirurgia geral. No quinto ano eram necessárias 15 semanas de OPD (departamento ambulatorial), seguidas do mesmo período de estudo em cirurgia geral. No sexto ano, os discentes faziam estágio rotativo de três meses cada, em cirurgia geral e pediatria, e os últimos três meses, em obstetrícia, especialidades cirúrgicas e pronto-socorro (Rockefeller Foundation, 29 set. 1959). Esse programa entrou em vigor em março de 1958, com a colaboração de funcionários que prestaram serviços sem remuneração, para que a reforma pudesse ser efetivada no início do ano letivo, com exceção do ensino de pediatria e neurologia, no terceiro ano, que ainda não havia sido completamente implementado.

O último financiamento para a EPM identificado na documentação foi uma doação de 131 mil dólares (Grant RF 61103), também para complementação salarial de pessoal em tempo integral (Rockefeller Foundation, 23 ago. 1961), aspecto considerado crucial para uma formação de médicos pautada em critérios de qualidade. Em análise sobre as implicações futuras do apoio oferecido à EPM, os membros da FR afirmavam que ela foi escolhida como elemento importante para o desenvolvimento futuro do programa de MEPH e que era possível que ela assumisse a liderança educativa em outra direção, se certas restrições legais fossem removidas: “Isso seria no sentido de tentar estabelecer uma escola de medicina que fosse uma ‘Escola Brasileira’, e não uma cópia de modelos importados” (Rockefeller Foundation, 29 set. 1959, p.22).

O fato de a EPM desenvolver um programa semelhante ao da Johns Hopkins, com currículo pré-médico e currículo médico concentrado, fazia a FR acreditar que aquela era uma imitação de um modelo exportado. A agência internacional não conseguia observar que, embora a escola brasileira estivesse alinhada com a proposta norte-americana naquele momento, desde antes do financiamento ela seguia caminho próprio, muitas vezes interagindo em zonas de contato com agentes nacionais e com profissionais de outras tradições científicas internacionais.

Nos últimos anos de vigência da cooperação, um dos itens avaliados negativamente pela FR foi o “fracasso” da EPM em assumir total responsabilidade sobre o salário dos funcionários. Em agosto de 1961 (prazo posteriormente estendido para 1962), os fundos fornecidos pela agência filantrópica internacional se esgotariam, o programa seria abandonado e, consequentemente, muitos funcionários seriam perdidos para outras instituições (Rockefeller Foundation, 23 ago. 1961). A liderança ocupada pela instituição brasileira levou a FR a permitir apenas mais um ano de apoio salarial, e o governo deveria encontrar fundo necessário para continuar o desenvolvimento da EPM.

Nem sempre o modelo de financiamento compartilhado, com a progressiva retirada do investimento da FR lograva êxito, especialmente porque, em algumas ocasiões, os governos locais não conseguiam ou não tinham interesse em assumir a responsabilidade sozinhos. No caso brasileiro, os primeiros anos da década de 1960 foram marcados por turbulência política que culminou na renúncia do presidente Jânio Quadros, em 1961, após apenas sete meses de governo; e pela posse de João Goulart, que sob duras acusações de comunismo foi deposto pelo golpe militar de 1964. As implicações dos contextos locais são fundamentais para se refletir sobre as cooperações internacionais. Exemplo disso pode ser observado em Batista (2024), que analisou as pressões de integrantes do governo nacional para a criação de uma escola de enfermagem em Niterói, com apoio do Serviço Especial de Saúde Pública, e que teve sérios problemas relativos ao local de funcionamento e à falta de estudantes.

## Considerações finais

A modernização do ensino médico na EPM é parte de processo mais amplo que envolveu instituições da América Latina e de diferentes lugares do mundo. O objetivo era criar departamentos de ensino e pesquisa que atendessem a determinados critérios, julgados importantes para ampliar a qualidade da educação, em um cenário de Guerra Fria, que acirrou a busca pela influência dos EUA e da URSS sobre os demais países do globo. A FR foi uma das instituições empenhadas nesse processo de modernização, sobretudo para conseguir demonstrar a viabilidade da sua metodologia de “efeito-demonstração”.

A EPM, por sua vez, era instituição de destaque no cenário nacional, com profissionais que mantinham uma rede internacional de trocas, o que chamou a atenção de membros da União Cultural Brasil-Estados Unidos e do *staff* da Fundação Rockefeller. O fato de a instituição ter sido federalizada, em 1956, parece ter contribuído para viabilizar os investimentos da agência filantrópica, mesmo que houvesse dúvidas sobre quais resultados seriam atingidos nessa aposta.

O dinheiro fornecido à EPM foi de grande importância para construção de prédios, compra de equipamentos e para tentar implantar regime de tempo integral em todos os departamentos, já que o trabalho em clínicas particulares “competia” com o trabalho na instituição financiada pela FR. O profissional idealizado deveria trabalhar com dedicação exclusiva. No máximo, em tempo integral geográfico, que permitia um número reduzido de atendimentos particulares, no mesmo equipamento físico onde atendia pela escola.

As bolsas disponibilizadas para que professores da EPM estudassem no exterior beneficiaram pesquisadores de destaque no campo paulista das ciências básicas, como José Ribeiro do Valle, José Leal Prado de Carvalho e seus assistentes. Um estudo detalhado sobre suas trajetórias internacionais pode auxiliar na compreensão das tensões existentes no processo de circulação do conhecimento, na formação profissional com financiamento da FR.

Por fim, o estudo dos financiamentos realizados pela FR à EPM demonstra que, entre meados de 1950 e 1962, a instituição brasileira conseguiu lograr amplo desenvolvimento com o auxílio da agência filantrópica internacional, mesmo que alguns objetivos ainda não estivessem consolidados, a exemplo da existência de fundos fixos para pagamento de pessoal em tempo integral, com apoio irrestrito do governo federal.

## Data Availability

Não estão em repositório.

## NOTAS

1 A Fundação Rockefeller surgiu em 1913, pela filantropia de John Rockefeller Jr., que acumulava riquezas com extração, refino e venda de petróleo nos EUA. Criou instituições de ensino sob a orientação do Relatório Flexner, publicado em 1910, realizou campanhas para erradicar enfermidades como a ancilostomíase, a febre amarela e a malária, e ofereceu bolsas de estudos para a formação de profissionais de saúde pública principalmente nos centros de ensino que apoiava.

2 Nessa e nas demais citações de textos em outros idiomas, a tradução é livre.
