# Supplementary material for: A modernização do ensino na Escola Paulista de Medicina e a Fundação Rockefeller, 1956-1962
Source: Hist Cienc Saude Manguinhos. 2025 Jan 10;31:e2024074. [Article in Portuguese] doi: 10.1590/S0104-59702024000100074 (PMC11723584; doi:10.1590/S0104-59702024000100074)
Supplement: Translation

# Translation: The modernization of medical training at Escola Paulista de Medicina and the Rockefeller Foundation, 1956-1962

**DOI:** 10.1590/S0104-59702024000100074en

**Authors:** Ricardo dos Santos Batista

**Affiliations:** i Professor, Post-graduate Program in History/Universidade do Estado da Bahia Alagoinhas – BA – Brazil. kadobatista@hotmail.com

**Keywords:** Escola Paulista de Medicina, Knowledge circulation, Rockefeller Foundation

## Abstract

The study analyses scientific knowledge circulation between Brazil and the United States, drawing on Rockefeller Foundation’s funding to Escola Paulista de Medicina to modernize medical training. The chosen initial chronological landmark is 1956, when the foundation started funding the Brazilian institution, and the final year is 1962, deadline for spending the grants. The study’s sources are dossiers collected at Rockefeller Archive Center, analyzed drawing on the evidential paradigm. The conclusion is that when the philanthropic agency decided to finance the school, the latter was already part of an international network of knowledge circulation, and its medical training modernization comprised investments in basic sciences, clinical practice, and the defense of full-time departments.

In *Relocating modern science: circulation and the construction of knowledge in South Asia and Europe, 1650-1900*, the Indian researcher Kapil Raj (2007) states that in the past decades, the alleged unity of modern practices of knowledge in the entire European space has been convincingly demolished. Instead of one sole “modern science,” now it is accepted that there are many traditions and dynamics of national and local knowledge spread through most of the Northern and Western Europe, with various modern agendas and intellectual influences. In Raj’s interpretation, he questions concepts such as “center,” which would produce the knowledge to be transplanted, and “periphery,” seen as the locus of passive reception of this knowledge (p.6). The author opposes the unidirectional ideas of dissemination and binarism present in the dichotomic interpretations and proposes using the concept of circulation, not as a mere transmission or communication of ideas, but rather as processes of encounter, power, resistance, negotiation and reconfiguration that occur in the interaction between cultures, by means of individuals who are “mediators” or local agents, who act in “contact zones” or in the interactions with other cultures. Based on these presuppositions, this paper aims to analyze the circulation of scientific knowledge between Brazil and the United States, between 1956 and 1962, by means of the funding by the Rockefeller Foundation (RF)^1^ to the Paulista School of Medicine (Escola Paulista de Medicina, EPM).

Before the creation of the World Health Organization (WHO) in 1948, the International Health Division (IHD) of the Rockefeller Foundation was the world’s most important philanthropic agency in public health. It received several names since its creation: International Health Commission (IHC), from 1913 to 1916; International Health Board (IHB), from 1916 to 1927; and from 1927 until its closure in 1951, it operated as IHD (Farley, 2004, p.2). IHB’s members followed the same philanthropic principles of John D. Rockefeller, a North American millionaire who invested in the international agency with the money originated from petroleum exploration, refining and trade, and advocated that philanthropy could not be mistaken for charity. Philanthropy should be understood as an investment provided to governmental agencies, and not to individuals, with a limited duration so as not become dependence, aimed at organizations committed to the continuity of the work after the end of the funding (Farley, 2004, p.3-5). Especially after the closure of IHD, the RF reduced the resources offered to public health projects and invested more widely in basic sciences, or laboratory sciences.

According to Gabriela Marinho (2004), the support of RF to researchers from EPM is a process still insufficiently analyzed and less known than the presence of the international agency at the University of São Paulo [Universidade de São Paulo, USP], for example. The author also states that, although the institution has been decisive in the constitution of a research standard, scarce and punctual references did not lead, yet, to the formulation of more consistent analyses on the impacts of its scientific production and the construction of a model of excellence regarding specific agendas. Thus, this analysis contributes to broaden the knowledge on the relationship between the international philanthropic agency and EPM, besides observing the knowledge produced in the Brazilian institution as part of a network of international knowledge. This paper draws close to interpretations presented in the book organized by Schneider (2002), according to which the RF’s philanthropic interests sought to reform themselves amid the complexity of local and wider influences.

The documents used to write this article were collected in loco at Rockefeller Archive Center, in May 2019, and contribute to the understanding of medical training modernization in Brazil as a complex and non-linear process. Among the institutions funded by RF with this purpose in the state of São Paulo, and whose documentation was consulted and digitalized, stands out the Ribeirão Preto School of Medicine [Faculdade de Medicina de Ribeirão Preto]. This might be the most emblematic case in Brazil in the 1950s, because since its creation it was expected to become “the Johns Hopkins of Latin America, the model modern medical school” (Barron, 17 dez. 1952a, p.1), in an explicit mention to the first North American university supported by RF in the early twentieth century (Marinho et al., 2024).

Also outstanding are the sources about EPM. For both institutions, there are dossiers related to the grants provided, comprising letters and correspondences; specific dossiers on materials purchased for departments, laboratories, libraries etc., and on professionals who were granted scholarships for studies abroad, aiming to modernize the training. Throughout its history in the twentieth century, RF believed that it could train leaderships or “key-elements” who would propagate the acquired knowledge when returning to their country of origin, and therefore it implemented a longstanding scholarship program, as demonstrated in the studies on scholarship holders’ trajectories by Batista (2019a, 2019b, 2020a, 2020b, 2020c, 2023); Batista and Silva (2020); Batista and Ferreira (2021, 2023); Batista, Porto and Ferreira (2023); and Batista and Mota (2021). In another perspective of analysis, prosopographic, one can find studies such as those by Korndörfer (2016, 2019) and Korndörfer and Ramacciotti (2021).

The sources used in this paper are composed of dossiers about EPM that aggregate, in addition to the above mentioned documents, also booklets that publicized the work carried out by the institution before the international agency’s support. The method of analysis used is the onomastic, which prioritizes the “name” as key-element in the archival research, through the evidential paradigm, which highlights the sources’ small details as significant signals for the analysis (Ginzburg, 1989a, 1989b).

## Medical training modernization and investment in basic sciences

Medical training modernization was an important issue for RF throughout the twentieth century. According to Elizabeth Fee (2016), by the end of the nineteenth century, public health was institutionalized in the USA’s municipal and state health secretariats, but few sanitarians had a specialized training in the area. Some were physicians, others engineers, lawyers, chemists, or biologists. In general, they were given part-time positions, paid for by political sponsorship, and could be promoted or removed from their positions based on political alliances and personal friendship. However, a new model of medical training emerged in the USA, consolidating resources in elite schools, drawing on the report produced by Abraham Flexner, in 1910, which changed the medical training in the country. Among the established changes were the recommendation for clinical experience in university hospitals controlled by a full-time faculty and for students’ familiarity with the laboratory (Cueto, Palmer, 2016).

In synchronicity with the Flexner Report, in 1914, a small group of public health leaders, gathered by invitation of RF’s General Education Board, identified the need to create formal institutions for the training of health professionals (Fee, 2016). Progressively, the investment of the international agency in medical and public health schools expanded to different places around the globe. As aforementioned, the first of them was the Johns Hopkins School of Hygiene and Public Health (Fee, 2016), followed by the School of Medicine and Surgery of São Paulo (Faculdade de Medicina e Cirurgia de São Paulo, FMCSP) (Batista, Mota, 2021). Among many other examples that could be mentioned, there were the investments in Edinburgh, where tradition and individualism were highly valued, but were considered by RF as an obstacle to its vision of medical reform (Lawrence, 2005); and East Asia, where there was funding for the Peking Union Medical College, through RF’s China Medical Board. During three decades, the institution was considered “the cradle of modern medicine in China” (Ryan, Bullock, 2017, p.22).

Regarding Latin America, between 1913 and 1940, RF expanded its interest in financing sciences in the physiology field in Peru and Argentina. Among the Argentinians, Bernardo Houssay, Nobel Prize laureate in 1947, directed the Institute of Physiology of the University of Buenos Aires (Instituto de Fisiología de la Universidad de Buenos Aires). According to Cueto (1994), RF’s presence in Latin America had three dimensions. First, by granting scholarships in the USA to medical students, it discouraged the preference of Latin American physicians for post-graduate training in European countries, especially in Paris. Second, because RF had deep doubts about the traditional schools of medicine, it promoted the establishment of new education and research centers independent from those institutions. And, third, the agency gave money to outstanding researchers in basic sciences. The donation of top-of-the-line equipment to institutions willing to guarantee places for full-time physiologists became one of RF’s main objectives (Cueto, 1994).

Rockefeller’s investment in the so-called basic sciences in Brazil was analyzed by Paloma Porto (2021), who studied the dynamics of the scientific funding policies at the Biological Sciences Institute (Instituto de Ciências Biológicas, ICB) of the Federal University of Minas Gerais (Universidade Federal de Minas Gerais, UFMG). The author discusses the program promoted by the international philanthropic agency with the objective of training basic sciences professors, directed in Latin America by Robert Briggs Watson. The RF stimulated microscale cognitive tendencies in ICB’s scientific researches, with emphasis on the handling of modern equipment and laboratory facilities, increasingly redirecting its investments to a “molecularization” of sciences (Porto, 2021).

In the early twentieth century, RF’s staff had visited several schools of medicine, and were shocked with the fact that most of the books in their libraries were in French. By 1950, they visited again the schools and noticed that most of the books were in English (Cueto, 2021). Other important changes operated through the first half of the twentieth century in Latin America were the integration of laboratory with clinics, and the rise of genetics, molecular biology and biophysics. In addition, it was noticed that there was an ideological rhetoric of scientific legitimation in a period marked by the efforts of North American administrations to contain the proliferation of communism (Cueto, 2021).

The modernization of medical training proposed by RF in the second half of the twentieth century occurred amid the Cold War conflict, in which the international technical cooperation was an outstanding element. According to Onofre Santos Filho (2005), in the bipolarity USA versus URSS, the ideas of development and modernization found a fruitful field for their propagation. In Point IV of the inauguration speech of the North American president Harry Truman (1945-1953), for example, he mentioned a new standard for the relationship with the so-called “underdeveloped” and “developed” countries. Drawing on this model, the relationship between the North and the South should be based on assistance provided by the developed countries, so that the underdeveloped countries would overcome their condition of misery and poverty. The rich countries, responsible for conducting this change, would not have abundant material resources to be transferred to the poor States, but would have technological knowledge that could be transmitted. In this logic, it would suffice that the latter followed the steps of the former, so they would reach the scientific and technological knowledge to transform themselves and get rid of poverty and ignorance, the only causes of the backwardness in which they were in. Still according to Santos Filho (2005), in addition to meeting the parameters of the new postwar order and the bipolar conflict, this conception of development allows to identify the agents of transformation: the newly-created international organizations, the national States and the modernizing elites.

In “Educational reform, modernization and development: a Cold War transnational process”, Óscar J. Martín García and Lorenzo Delgado Gómez-Escalonilla (2020) affirm that the USA were a leading force behind the processes of educational reform in the Cold War. In the 1960s, the US administration started to show greater interest in education in its relations with the countries of the so-called global “periphery” and “semi-periphery.” In September 1961, the report entitled “International Educational and Cultural Policies and Programs for the 1960s” collected the proposals of several working groups gathered by John Kennedy’s administration (1961-1963), with the purpose of elaborating “a philosophy and objectives for educational, cultural and scientific activity for the decade of the sixties as they relate do both governmental and private sectors. According to this report, education was a basic ingredient of the early stages of economic development” (García, Gómez-Escalonilla, 2020, p.14).

The authors also stress that the push toward modernization of the countries considered as backward would involve, through modern educational systems, the training to create human capital with the necessary technical capacities to resolve the underdevelopment problems, especially in international programs of education, culture and science. However, the educational programs were not conducted solely by governmental actors. The United Nations Educational, Scientific and Cultural Organization, the Organization for Economic Cooperation and Development, and the World Bank, for instance, were also interlocutors of educational reform. In this context, RF intensified its funding actions for medical training modernization, as was the case of EPM.

## The EPM and full-time departments

The creation of EPM occurred in a context favorable to the expansion of medical training in São Paulo. Since 1891, the legislation of São Paulo had already provided for the creation of a school of medicine, but the FMCSP was created in 1913 (Mota, 2005). The FMCSP underwent changes in the Pedro Dias reform, in 1924, and in the reorganization promoted by Decree 5.351, of 16 January 1932, with the objective of adapting to the changes imposed by the Francisco Campos educational reform. In 1933, there was a high number of applicants for the FMCSP entry exam and, even though 119 candidates achieved the average score to enter the pre-medical school, there were only seventy vacancies available. According to Márcia Regina Barros da Silva (2003), there was dissatisfaction from those who exceeded the vacancies and they began to request the increase of the number of places. However, those aspiring to become physicians did not imagine the intention of another group, composed of professors who wished for a new space for medical training in São Paulo. The convergence of interests between the two groups would be fundamental to the creation of EPM.

Hence, in 26 June 1933, the new São Paulo medical institution was created (Figures 1 and 2). Its founders were linked to three broad areas of work: the educational practice, at the Holy House of Mercy (Santa Casa de Misericórdia); the clinical practice in sanitary services of the state of São Paulo; and the activities conducted in research institutes (Silva, 2003). The current historiographic production affirms that EPM’s history can be divided in two phases. The first phase comprises from the installation of the medical course, in 1933, until the beginning of the construction of the university hospital, named Hospital das Clínicas. The second phase begins with the graduation of the first class, in 1938, and ends with the federalization of the school, in 1956 (Silva, 2003). This article aims to help in the understanding of aspects related to what can be considered the third phase, characterized by the funding provided by RF to EPM, between 1956 and 1962.

**Figure 1:** : Escola Paulista de Medicina (Source: EPM, 1951, p.12)

**Figure 2:** : Students at Escola Paulista de Medicina (Source: EPM, 1951, p.17)

In the years before RF’s funding to the Brazilian institution, the Brazil-United States Cultural Union [União Cultural Brasil-Estados Unidos], affirming that it fulfilled its role of promoting knowledge and understanding between the two countries, wrote to the philanthropic agency’s staff with the aim of drawing attention to a medical organization in São Paulo, which deserved to be better known due to its efficiency and scientific honesty: the EPM (Amorim, 5 jul. 1951). The correspondence written by the director-general, Rone Amorim (5 jul. 1951, p.1), stated that the school had already achieved a privileged reputation for keeping high standards of education; therefore, he suggested a visit from a Rockefeller member, when in São Paulo, which would ensure a “mutually beneficial” contract. In reply, Robert Morison, assistant director of the Division of Natural Sciences at RF, thanked for the correspondence, informed that some members of the agency already knew the school and hoped that soon they would be able to visit São Paulo again.

One of the members assigned to this observation was Oliver Jones (27 out. 1952), whose opinion was contrary to funding EPM. He wrote to Harry Miller Jr. and expressed having been quite impressed with the research activities in São Paulo, but was not impressed with the way in which the schools of medicine were managed. He doubted that it would be possible to raise the medical standard or the quality of general clinics, unless something drastic was done about the “admission policy to the school and an elimination of the weak and poorer students after the second or even third year of medical school” (Jones, 27 out. 1952, p.1). The main criticism to EPM was that it was very much dependent on students’ monthly fees to function and as a result it admitted classes with more students than it could accommodate in its facilities and with the existing staff. At the same time, being a private institution allowed for a more progressive attitude in relation to appointments and promotions of professors (Jones, 11 nov. 1952).

After a little more than a month, another RF’s staff member, E.S. Guzman Barron, who had also visited the institution, showed enthusiasm with EPM’s director, Jairo Ramos, and the biochemistry professor Leal Prado. According to Barron (17 dez. 1952b, p.1), the latter, defined as a former physiologist anxious to fully dedicate himself to biochemistry, had been one of the main factors for the conversion of the project in Ribeirão Preto from “a sort of São Paulo nursery into a modern medical school.” After considering the evaluations, RF decided to invest in EPM.

The first funding (Grant RF 50652) was granted for the development of the institution in the city of São Paulo in April 1956, with the amount of 105 thousand US dollars to be used until 30 June 1958, which was extended until November of the same year. The grant was provided after doctor Bugher, a RF staff member, presented a project that defined the assistance for professional training in Medical Education and Public Health, as well as for medical and biological research (Rockefeller Foundation, 4 abr. 1956). The aim of RF’s support was to integrate EPM into its basic program for the improvement of medical training and research in Brazil, by means of the supply of training facilities for the implementation of scholarships and research material:

In spite of limited financial resources and clinical facilities inferior to those of the Faculty of Medicine of São Paulo, the Paulista School has, from its inception, enjoyed a reputation of being among the better medical schools of Brazil. While it has lacked depth in teaching staff and subordinate technical personnel, particularly in the preclinical departments, its department heads and their principal assistants make up a group of teachers and research workers equal to that of any other Brazilian school. Some of these professors are the best representatives of their professional disciplines that Brazil can present and this proposed appropriation is designed particularly to support their departments (Rockefeller Foundation, 4 abr. 1956, p.2-3).

Although in this document there is a negative note regarding the scarcity of professionals, before receiving the grant from RF the EPM staff comprised individuals who would later become references in the national scientific production. In 1951, worked at the institution: André Dreyfus, chair of General Histology and Embryology; Antônio Carlos Pacheco e Silva, Psychiatric Clinics; and Walter Pereira Leser, chair of Hygiene. The institutional development of EPM had been boosted by the support received from the Health Department of the state of São Paulo, the Direction of EPM, and, from 1952 onward, the National Council of Research, which contributed to the successful activities of the Pharmacology and Biochemistry laboratories. Regarding the students, by the end of the school year of 1954, the institution had already graduated 1,366 physicians, of which 402 were from the city of São Paulo, 494 from the state of São Paulo, 82 from the states of Paraná, Rio Grande do Sul, Minas Gerais and Federal District, 34 from several other states and 17 from foreign countries such as Canada and the USA (Rockefeller Foundation, 4 abr. 1956).

A report produced to celebrate its existence from 1948 to 1953, shows data that help to identify some of the professionals who composed the network of production and circulation of scientific knowledge in EPM (Chart 1).

**Chart 1:** : Staff members of the Biochemistry and Pharmacology laboratories, 1948-1953

Name	Background
José Ribeiro do Valle	Full professor of Pharmacology, former assistant at Butantan Institute [Instituto Butantan], physician of the Health Department of the State of São Paulo
José Leal Prado	Full professor of Physiological Chemistry, former assistant at Butantan Institute, physician of the Health Department of the State of São Paulo
Zuleika Pentone Picarelli	Chemist from the School of Philosophy, Sciences and Letters of the University of São Paulo, first assistant of the Chair of Pharmacology, with full-time dedication thanks to support from the Nacional Council of Research
Eline S. Prado	Chemist from the School of Philosophy, Sciences and Letters of the University of São Paulo, Master of Sciences from the McGill University in Canada, first assistant in Physiological Chemistry, with full-time dedication thanks to support from the Nacional Council of Research
Antônio Cechelli de Mattos Paiva	Physician from Paulista School of Medicine [Escola Paulista de Medicina], second assistant of the Chair of Physiological Chemistry, with full-time dedication, scholarship holder in 1953 of Doutor Joaquim Libânio Leite Ribeiro Scholarship
Catharina M. W. Brandi	Chemist from the School of Philosophy, Sciences and Letters of the University of São Paulo, third assistant of the Chair of Physiological Chemistry, scholarship holder of Lordelle Laboratories [Laboratórios Lorderle]
Bella Regina Kupper	Pharmaceutic, supernumerary assistant, with full-time dedication

Source: Rockefeller Foundation (1948-1953).

In 1954, EPM’s curriculum had also been drastically reorganized. The changes were approved by the National Council of Education [Conselho Nacional de Educação] and came into effect in 1955. RF’s members were of the opinion that the school’s organization was comparable to the schools in the United States, regarding training, and that the main discrepancies were imposed by Brazilian legislation, e.g., the need of pre-medical education and certain legal requirements (Rockefeller Foundation, 4 abr. 1956). Three basic sciences chairs were occupied by outstanding full-time professors and researchers: Biochemistry, by José Leal Prado; Pharmacology, by José Ribeiro do Valle; and Microbiology, by Otto Bier. As from 1956, each of these chairs should receive budget from the National Council of Research to complement the wages of auxiliaries and technicians, so that the staff of the three departments would work full-time.

From 1 March 1956 onward, two chairs (Biochemistry and Pharmacology) started to operate in a new building for education, research and shared use of equipment. The Pathology chair, also occupied by a full professor, Moacyr de Freitas Amorim, requested support from the National Council of Research in 1956, and constructed another building for the disciplines related to its area (Rockefeller Foundation, 4 abr. 1956). Between 1957 and 1958, this department was expanded and occupied rooms that were fully renovated and reequipped (Rockefeller Foundation, 29 set. 1959).

EPM’s laboratory researchers maintained close contact with national and international researchers through correspondence, exchange of bibliography and mutual visits. Among the institutions with which they communicated were the Schools of the University of São Paulo, School of Medicine of Belo Horizonte (Laboratories of Anatomy, Pathology, Pharmacology and Physiological Chemistry), School of Medicine of Recife (Álvaro Ozório de Almeida Physiology Institute), School of Medicine of Paraná (Physiology and Pharmacology Laboratories), National School of Medicine, Oswaldo Cruz Institute, Butantan Institute, among others (Rockefeller Foundation, 1948-1953).

EPM’s laboratories had already been visited by R.A. Lambert, Alan Gregg, W.W. Oliver and Harry Miller Jr., all RF staff members; Luiz F. Leloir and R. Caputo, from Buenos Aires; E. Rothlin, from Basel; A.L. Tatum, from Madison, and A. de Barbieri, from Milan. Finally, among the 17 researchers who held conferences at EPM, there were names such as Bernardo A. Houssay, director of the Institute of Biology and Experimental Medicine of Buenos Aires [Instituto de Biología y Medicina Experimental de Buenos Aires]; Frank G. Youn, professor of Biochemistry at Cambridge University; and J.M. Carlisle, director of the Medical Division of Merck, United States, besides Earl Walker, professor of Neurosurgery at Johns Hopkins University (Rockefeller Foundation, 1948-1953).

It can be stated, therefore, that EPM’s researchers maintained relationship with the large centers of medical knowledge worldwide and emerged within a complex network of knowledge exchanges, since the early 1950s. The funding proposed by RF in 1956 followed the diagnosis made by Steven Palmer (2015) about the method of action of the international agency, whose interest was in institutions and countries that already developed some type of action in the area in which it intended to invest. Since its creation, RF supported governmental institutions that after a period of funding would be able to assume the responsibility of the actions developed and serve as example for the replication of its model (Marinho, 2013).

The support of RF to EPM was meant for the purchase of equipment for graduation education and research in the departments of Biochemistry, Pharmacology and Microbiology, and to make feasible the reception of national and international students for post-graduation. It was expected that these three departments would become important centers for the training of Brazilian medical schools’ professors, in the scope of a project that at the time was funded by the Coordination for the Improvement of Higher Education Personnel [Coordenação de Aperfeiçoamento de Nível Superior, Capes] (Rockefeller Foundation, 4 abr. 1956). The recommendation was that the resources provided by RF were distributed as follows: 52.4% for basic sciences (of which 33% exclusively for Biochemistry, Pharmacology and Microbiology) and 47.6% for Medical Clinics (Figure 3).

**Figure 3:** : Distribution of resources, Grant RF 56052 (Source: Rockefeller Foundation, 4 abr. 1956, p.5)

The distribution of resources kept a certain balance between the financed areas, demonstrating the priorities of RF and EPM in the process of training modernization. It is noticeable that the Medical Clinics was boosted especially due to the performance attained by Professor Jairo Ramos.

On 3 December 1958, a new grant of six hundred thousand US dollars (Grant RF 58217) was approved, aiming to ensure the consolidation and continuation of EPM’s development, with the change of all departments into a full-time training and research regime. This grant should be used in a period of minimum three years and maximum five years, as from 1 January 1959 (Rockefeller Foundation, 3 dez. 1958). The good results of the investment at EPM led to the approval in 1959 of a new grant of U$ 633.500,00 (Grant RF 58...), with the same objective of consolidating the school’s development, until December 1961:

The remarkable progress of the school during the past two years was not forseen. This progress suggests that with further, and broader, support the school may develop into a position of pre-eminent leadership in Brazilian medical education. This prospect seems in itself and in relation to development of program in Brazil, to justify favourable consideration of the request of the school`s administration, endorsed by the federal Ministry of Education, for such support (Rockefeller Foundation, 29 set. 1959, p.2).

It is worthy of note that the first grant provided to EPM was a bid on the potential of its development, but there was no exact dimension of the future implications of the action. The institution had been incorporated into the federal educational system only ten weeks before, and the remaining year of 1956 was used to transfer its assets to the Union, except for the São Paulo Hospital [Hospital São Paulo, its university hospital], and to fulfil other legal formalities.

For RF, the quality of EPM’s professors was improved through scholarships and travel grants. The Biochemistry professor, e.g., spent one year in France and the United States, and his main assistant spent two years in the United States with a scholarship. Their wives were both also trained in biochemistry, worked as auxiliaries in the department, accompanied them abroad, and it was believed that, to some extent, they benefited professionally from their trips (Rockefeller Foundation, 29 set. 1959). Gender asymmetries, that are socially and culturally constructed, not rarely prevented scientists’ wives from being granted their own international scholarships. Therefore, the wives had to make good use of travelling as their husbands’ companions, try to produce knowledge and, whenever possible, implement research agendas in international institutions.

Still regarding EPM’s scholarship holders, two scholarships of the Medical Education and Public Health (MEPH) program were effective for assistants of the Department of Medicine: in 1959, one scholarship holder returned after two years of training, and another after one year. Finally, the presence of eight scholarship holders at the school with grants from Capes and RF, mostly in the departments of basic sciences, provided pedagogical strength, preparing them for their respective positions in Brazil and Chile (Rockefeller Foundation, 29 set. 1959).

The Brazilian government also contributed with approximately three hundred thousand dollars, in 1957, and 303 thousand dollars, in 1958. It provided approximately five million *cruzeiros* (the Brazilian currency of the time) to conclude the new Biochemistry and Pharmacology building, reconstruct the Department of Microbiology and start the new library building (Rockefeller Foundation, 29 set. 1959). However, the operational budget only provided funds for part-time jobs for professors. In order to make feasible the full-time jobs for the “key-elements” of research, the National Council of Research allocated 384 thousand *cruzeiros* in 1957 and 923,500 *cruzeiros* in 1958.

A department that was under the expectation of RF during the entire funding was that of Medical Clinics. It was expected not only to improve the graduate and post-graduate training, but also to supply medical research equipment, such as the laboratory diagnosis equipment for the use of students in the medical cases attributed to them. Jairo Ramos, professor and head of that department, was considered by RF staff as an outstanding physician and educator, who was also a former director of the school. It was believed that two events helped to crystalize the role he performed in the reform of medical training in São Paulo: the Seminar on Medical Education held in Ribeirão Preto, in 1956, and the contact with the School of Medicine of Cali, in an expedition of EPM lead by him.

Finally, the frequent contact of Ramos with several members of RF, as Dana W. Atchley, Clifford Grullee, Robert Shank, Robert Glazer, George Reader and William B. Wendel, since 1956, was useful for the formulation of plans for EPM (Rockefeller Foundation, 29 set. 1959). About Ramos, it was affirmed that together with other progressive elements of the Brazilian medical training, he was the one responsible for breaking from tradition, perhaps more than any other person, in an effort to modernize medical education in Brazil. Ramos and his team of assistants, eight of them having been trained in the United States, spent over half their time in teaching activities (Rockefeller Foundation, 4 abr. 1956).

The main aspects of the plans achieved by Jairo Ramos for EPM, with the help of physicians from RF, were: the creation of full-time basic science and the establishment of geographic full-time in the main clinical departments. According to Voltarelli (1997, p.152), in this perspective, most teachers would receive additional wages for assistance services provided to the university hospital, and would have the opportunity to assist private or insured patients and have paid shifts without leaving their institutions, which would configure a full-time geographic regime.

Still on this working regime, according to Martins (2000), the Ribeirão Preto School of Medicine, of the University of São Paulo, was the first one created in the interior of the country, entirely with public resources, and required full-time dedication from all professors as a way to foment research. At that time, it was already recognized the little attraction exerted by the salaries in areas of application; therefore, the practice of private clinics was admitted, in a controlled manner and a determined amount of time per week, in the premises of the same unity (from a total of 750 hospital beds, only 12 beds, or 1.6% of the total, were intended for this purpose). This was then the so-called full-time geographic dedication. Free housing was also provided, in addition to full retirement.

In 1959, the Department of Medical Clinics of EPM had 18 physicians. Ten of them, including Jairo Ramos and associate professors, received part-time wages, and the other eight were voluntary professors, with nominal appointments in the faculty. It was considered that five of these eight voluntary, non-paid, staff were “amongst the best youmg elements in Brazilian medical education, four trained in the United States and one, the cardiologist, in the Institute of Cardiology in Mexico” (Rockefeller Foundation, 29 set. 1959, p.6). Ramos proposed to ensure permanent appointments for those five men.

There was also the wish for the development of a training program to prepare the technical staff in Brazil and abroad; a complete reorganization of the medical library and the construction of a special premise to house it, in 1959-1960; the construction of a building for physiological sciences, in 1960-1961, and a renovation of the existing spaces for the expansion of departments associated to morphology (Rockefeller Foundation, 29 set. 1959).

Among all these aspects, the one that was seemingly more outstanding was the adoption of full-time work:

Geographic fulltime has been put into effect at the state medical school at Ribeirão Preto and one department (Clinical Medicine) at federal faculty of medicine in Salvador (Bahia) has fulltime (exclusive dedication). But this is the first instance in Brazil where a federal faculty of medicine proposes to stablish as rapidly as possible, geographic fulltime throughout all its clinical departments. Only through the establishment of this administrative discipline can the proposed reformation is essential: it is basic to any real improvement of medical education in this or any other school in Brazil (Rockefeller Foundation, 29 set. 1959, p.8).

The staff of RF was thrilled about the experience developed at EPM because, among other reasons, it was a federal institution, which seemed to provide safer bases for the project’s achievement. The Ribeirão Preto School of Medicine belonged to the state of São Paulo, and at the School of Bahia, the full-time geographic work was implemented in only one department, which did not fulfil satisfactorily the expectations of training modernization in a medical institution.

The concept of medical clinics reform proposed by EPM was considered simple: the students would receive a broad training in the area as soon as possible in the curriculum, and should remain under the supervision of the competent professors during the entire contact with patients. It was stated that most Brazilian medical students had no activity after the single class shift, remaining aimless after midday, left to their own plans, and that their training, even in the best of circumstances, tended to be fragmented, lacking coordination, being inefficient and ineffective (Rockefeller Foundation, 29 set. 1959).

The EPM proposed that it would initiate the students’ clinical training in the hospital one year before the curriculum, in comparison to what occurred in other schools. According to the education program, in the third year there would be 120 hours (in 15 weeks) of classes on methods of physical diagnosis in medicine, surgery, neurology and pediatrics, with 100 hours (10 weeks) of performance and interpretation of clinical laboratory tests. The fourth year would have a minimum of 660 hours (in 15 weeks) of clinical internship in general medicine, including neurology and pediatrics, followed by 15 weeks of general surgery. In the fifth year, were necessary 15 weeks of OPD (outpatient department), followed by the same study period on general surgery. In the sixth year, the students would have a rotatory internship of three months each, in general surgery and pediatrics, and the last three months in obstetrics and emergency care (Rockefeller Foundation, 29 set. 1959). This program came into effect in March 1958, with the collaboration of staff who provided services without payment, so that the reform could become effective at the beginning of the school year, except for pediatrics and neurology training, in the third year, which had not yet been fully implemented.

The last funding to EPM identified in the documentation was a donation of 131 thousand dollars (Grant RF 61103), also to complement the wages of full-time personnel (Rockefeller Foundation, 23 ago. 1961), an aspect considered crucial for medical training based on quality criteria. In an analysis on future implications of the support provided to EPM, the members of RF stated that it was chosen as an important element for the future development of the MEPH program and it was possible that it would take on the leadership of training in another direction, if certain legal restrictions were removed: “This would be in the the direction of trying to stablish a school of medicine which would be a ‘Brasilian School’ rather than a copy of imported models” (Rockefeller Foundation, 29 set. 1959, p.22).

The fact that EPM developed a similar program to that of Jonhs Hopkins, with a pre-medical curriculum and a concentrated medical curriculum, led RF to believe that it was an exported model. The international agency was not able to notice that although the Brazilian school was at that moment aligned with the North American proposal, EPM followed its own path before receiving the RF funding, often interacting in contact zones with national agents and professionals from other international scientific traditions.

In the last years of the cooperation, one of the items negatively assessed by RF was the EPM’s “failure” in assuming full responsibility over the staff’s wages. In August 1961 (deadline later extended to 1962), the funding provided by the international philanthropic agency would be extinguished, the program would be abandoned, and consequently many staff members would be lost to other institutions (Rockefeller Foundation, 23 ago. 1961). The leadership of the Brazilian institution led the RF to allow only one more year of support for wages, and the government should find the necessary funds to continue EPM’s development.

The model of shared funding with the progressive withdrawal of RF’s investment was not always successful, especially because in some occasions the local government was not able or had no interest to assume the full responsibility. In the Brazilian case, the early years of the 1960s was marked by political turbulence that culminated in the resignation of president Jânio Quadros, in 1961, after only seven months in office; and by the inauguration of João Goulart, who under harsh accusations of being a communist was overthrown by the military coup in 1964. The implications of local contexts are crucial for the reflection on international cooperation. An example can be observed in Batista (2024), who analyzed the pressure of members of the national government for the creation of a nursing school in Niterói (state of Rio de Janeiro), with the support of the Public Health Special Service [Serviço Especial de Saúde Pública], which had serious problems related to the place of operation and the lack of students.

## Final considerations

The modernization of medical training at EPM is part of a broader process involving institutions of Latin America and different places worldwide. The aim was to create training and research departments that would fulfil certain criteria considered important to enhance the quality of education, in a context of Cold War that intensified the search for influence by the United States and the USSR over the other countries of the world. The RF was one of the institutions engaged in this modernization process, especially to demonstrate the viability of its “demonstration-effect” methodology.

The EPM, on the other hand, was an outstanding institution in the national context, with professionals who maintained an international exchange network, which called attention of members of the Brazil-United States Cultural Union and of the Rockefeller Foundation staff. The institution’s federalization in 1956 seems to have contributed to enable the philanthropic agency’s investments, even though there were doubts about what results could be achieved in this bid.

The funding granted to EPM was highly important to build facilities, purchase equipment and seek to transform all departments into full-time, since the work in private clinics “competed” with the job in the institution funded by RF. The idealized professional should work under exclusive dedication; at most, in geographic full-time, which allowed a reduced amount of private assistance in the same physical premise of the assistance provided to the school.

The scholarships granted to EPM’s professors to study abroad benefited outstanding researchers from São Paulo in the field of basic sciences, such as José Ribeiro do Valle, José Leal Prado de Carvalho, and their assistants. A detailed study on their international trajectories can add to the understanding of existing tensions within the process of knowledge circulation in the professional training with funding from RF.

Finally, the study of funding provided by RF to EPM shows that from the mid-1950 to 1962, the Brazilian institution achieved a broad development with the support of the international philanthropic agency, even though some of the objectives had not been consolidated, e.g., the availability of fixed funds for the payment of full-time personnel with unrestricted support of the federal government.

## Data Availability

Not deposited in a data repository.

## NOTES

1 The Rockefeller Foundation was created in 1913, by the philanthropy of John Rockefeller Jr., who accumulated wealth with the extraction, refinery and trade of oil in the United States. It created educational institutions under the orientation of the Flexner Report, published in 1910, carried out campaigns to eradicate diseases such as ancylostomiasis, yellow fever and malaria, and granted scholarships for the training of public health professionals, especially in the educational centers that received its support.
